# Supplementary material for: Size control in mammalian cells involves modulation of both growth rate and cell cycle duration
Source: Nat Commun. 2018 Aug 16;9:3275. doi: 10.1038/s41467-018-05393-0 (PMC6095894; doi:10.1038/s41467-018-05393-0)
Supplement: Supplementary file 1 — Supplementary Information [file 41467_2018_5393_MOESM1_ESM.pdf]

Size control in mammalian cells involves modulation of both growth rate and cell cycle duration

Cadart et al.

Supplementary Information

Supplementary Note 1: Mathematical Framework ………………………………………..1

Supplementary Figures 1-7……………………………………………………….…….……….8

Supplementary References ………………………………………………….…………22

Supplementary Note 1: mathematical framework to compare size homeostasis mechanisms in various organisms

1 Brief Introduction

The correlation pattern between measured variables such as cell sizes and growth rate across well-defined cell-cycle intervals and the durations of such intervals contains a wealth of information about the underlying homeostatic mechanisms[1].

The general patterns of such correlations, as well as the links to the molecular mechanisms are still incompletely understood. A commonly accepted hypothesis in both bacteria and yeasts is that size control relies on an adaptation of cell cycle duration to growth, typically through either a critical size threshold ("sizer") or a critical added size threshold ("adder") (reviewed in[2–6]). Indirect evidence[3,7–9] as well as recent direct evidence[10] supports this "timing adaptation" hypothesis for mammalian cells and is reinforced by our direct observation of a negative correlation between G1 duration and volume at birth (Main text, Fig. 4c-d).

The complementary hypothesis that the speed of growth is coupled to cell cycle[11] and can be actively modulated to maintain size control[12] is comparatively less explored. This is due to the fact that growth has in many cases been shown to be well described by a mono-exponential curve, in *E. coli*[13–16], and *S. cerevisiae*[15,17,18] single cells, and also in mammalian cells[15,19–21]. We recall here the terminology adopted in the Main Text for growth speed, which is defined as the time derivative of size, and for growth rate, which is the derivative of growth speed as a function of instantaneous size (hence an inverse time, see Supplementary Fig. 7a). However, recent studies of mammalian cells also hypothesized a control of cell-cycle progression relying on modulations of growth speed, either based on direct measurements[22] or indirectly[19,23]. We find that single cells grow on average exponentially, in the sense that the conditional average of the growth speed for cells of a given size increases fairly linearly with size. We also report that this positive trend between growth speed and volume (thus the approximately constant growth rate) is smaller for cells that are larger at birth, and larger for those that are born smaller (see Fig. 6e and the discussion of our experiments on Roscovitine-treated HeLa-hgem cells).

To investigate the interplay between these the two distinct mechanisms of size homeostasis, modulation of timing and growth rate, we developed a mathematical framework and used it to quantify their relative contributions to cell size control in experimental data. The following sections describe this approach, which is in principle applicable to both the whole cell cycle and to specific cell-cycle stages.

2 Model ingredients

Motivated by the fact that linear correlations are able to explain most patterns in existing data we follow the framework of refs.[24,25], where size homeostasis is represented as a linear correction to perturbations in logarithmic size and exponential growth rate. Each cell is born with a certain size. Its interdivision time or cell-cycle stage duration has an intrinsic stochasticity and may be chosen based on initial size. Its growth is represented as an exponential growth rate α and a cell-cycle (or cell-cycle subperiod) timing τ, which may be coupled to size. Under these

assumptions, the final volume (in a cell cycle or subperiod) is represented as the result of a size-dependent growth and timing

$$V_f = V_0 \exp\left[\alpha(V_0)\tau(V_0,\alpha)\right] \ , \tag{1}$$

where V_x is volume (see also Table 1 for variable definitions). Here, α and τ quantify the specific growth rate and cell cycle (or interval) duration and they are both random variables. For convenience, we define $q_x = \log(V_x)$ and consider the fluctuations of logarithmic size. The overall multiplicative growth of a cell in one cycle is quantified by $G = q_f - q_0 = \log \frac{V_f}{V_0} =: \alpha\tau$.

Note that this framework does not formally require the hypothesis of exponential growth, but just states that growth during a cell cycle or subperiod is represented as a product of the time interval and an effective (exponential) growth rate. However, for cells that grow sub-exponentially, the variable α carries a natural dependency on τ which makes interpretation more complex. In brief, for non-exponential growth rate, the contribution of growth rate to size-homeostasis may be a sum of two components, (i) an active size-based compensation of the growth speed, for example smaller cells at a certain checkpoint decide to accelerate, etc. and (ii) a passive basal mode of growth, e.g. linear growth (see section 5 below, which explicitly addresses this question within the modeling framework).

Variable	*Description*	*Definition*
t	time	
V_0	volume at birth	
$V(t)$	volume at time t	
V_f	volume at division	
τ	time at division	
α	growth rate	see Eq. 1
$\langle\alpha\rangle$	average growth rate	
δ_α	growth rate fluctuations	$\delta_\alpha = \alpha - \langle\alpha\rangle$
q_0	logarithm of the initial volume	$q_0 = \log(V_0)$
$\langle q_0\rangle$	average of q_0	
σ_q^2	variance of q_0	
q_f	logarithm of the volume at division	$q_f = \log(V_f)$
δ_q	initial volume log fluctuations	$\delta_q = q_0 - \langle q_0\rangle$
G	overall multiplicative growth	$G = q_f - q_0 := \alpha\tau$
φ_τ	relative contribution of δ_q to fluctuations of the divison time	see Eq. 2
T_τ	relative contribution of δ_α to fluctuations of the divison time	see Eq. 2
φ_G	relative contribution of δ_q to fluctuations of G	see Eq. 3
T_G	relative contribution of δ_α to fluctuations of G	see Eq. 3
γ	contribution of δ_q to fluctuations of the growth rate	see Eq. 4
λ	slope of the size-growth plot	see Eq. 5
θ	contribution of δ_q to fluctuations of the division time	see Eq. 6
β	exponent of polynomial growth	see Eq. 8

Table 1: Table of the main variables

3 Relative roles of timing and growth in size homeostasis

This section shows how the homeostatic control strength on overall growth G is a balance of size-based correction by timing (i.e., on τ) and correction by growth rate modulation (i.e., on α).

Introducing the notation $\delta_q = q_0 - \langle q_0\rangle$ to represent the initial size fluctuations of a given cell, and $\delta_\alpha = \alpha - \langle\alpha\rangle$, which represents the fluctuations in growth rate, we can write the overall

timing as a function of these two fluctuations,

$$\tau - \langle \tau \rangle = -\langle \tau \rangle \left(\varphi_\tau \delta_q + T_\tau \delta_\alpha \right) + \nu_\tau \ , \tag{2}$$

stating that the duration of the time interval could be modulated based on deviations from the mean size. Positive values of the coupling constants φ_τ and T_τ indicate a homeostatic correction (e.g. a cell born smaller growing for a longer time), while negative ones stand for noise-increasing ones. These variables quantify the size-based and specific growth-rate based modulation of overall period timing; they are measurable from conditional averages or correlations, similarly to the slope of the size-growth plot. Finally, ν_τ represents the residual noise on the period timing, when the dependencies on the other variables are removed.

Equally,

$$G - \langle G \rangle = -(\varphi_G \delta_q + T_G \delta_\alpha) + \nu_G \ , \tag{3}$$

where analogous variables and coupling constants have been defined (see Table 1). Since $G = \alpha\tau$ and $\alpha = \langle \alpha \rangle + \delta_\alpha$, the two conditions on timing and overall growth have to be related. We can write the overall growth as

$$G = \langle \alpha \rangle \tau + \tau \delta_\alpha = \langle \alpha \tau \rangle - \langle \alpha \rangle \langle \tau \rangle \varphi_\tau \delta_q + \langle \tau \rangle (1 - \langle \alpha \rangle T_\tau) \delta_\alpha + ... \ ,$$

where we have neglected nonlinear terms and implied noise terms. The above equation gives an equivalence between the coupling parameters for G and for τ, in absence of any control on the growth rate α.

$$\varphi_G = \langle \alpha \rangle \langle \tau \rangle \varphi_\tau$$

and

$$T_G = -\langle \tau \rangle (1 - \langle \alpha \rangle T_\tau) \ .$$

We now deal with the fact that the growth rate fluctuations can also be linked to size fluctuations (Fig. 7c), i.e.,

$$\alpha - \langle \alpha \rangle = -\langle \alpha \rangle \left(\gamma \delta_q \right) + \nu_\alpha \ . \tag{4}$$

For exponentially growing cells, this equation expresses the fact that for positive values of γ cells that are born (or enter a cell-cycle stage) larger than average can correct their sizes by growing with a slower growth rate, and cells that are born (or enter a cell-cycle stage) with a smaller size than average can correct by growing at a faster growth rate. Conversely, for negative values of γ, growth rate variability increases systematically size variability. For sub-exponential growth, these correlated fluctuations can be homeostatic, but they cannot be interpreted as an active size-based correction. The well-known case is that linearly growing cells without any timing correction (i.e., a timer), perform size correction and add an average constant size at every cycle[26]. A more general case interpolating parametrically linear and exponential growth is discussed in the following section. Nevertheless, our formalism remains valid, and gives a decomposition of size correction into separate contributions due to modulation of timing and of the growth rate.

The slope λ of the size-growth plot is computed considering the conditional average of G over logarithmic size q (Fig. 7a),

$$\langle G \rangle_q = \langle G \rangle - \lambda \delta_q \tag{5}$$

Computation of this average gives

$$\langle G \rangle_q = \langle G \rangle - \varphi_G \delta_q - T_G \langle \delta_\alpha \rangle_q = \langle G \rangle - \left(\varphi_G - \gamma \langle \alpha \rangle T_G \right) \delta_q$$

leading to the relationship

$$\lambda = \varphi_G - \gamma \langle \alpha \rangle T_G$$

In a similar way, if we define the control parameter θ as the slope of the plot of τ *vs* logarithmic

initial size (Fig. 7b), i.e.,

$$\langle\tau\rangle_q = \langle\tau\rangle - \langle\tau\rangle\theta\delta_q \tag{6}$$

we have that:

$$\langle\tau\rangle_q = \langle\tau\rangle - \langle\tau\rangle\varphi_\tau\delta_q - \langle\tau\rangle T_\tau\langle\delta_\alpha\rangle_q = \langle\tau\rangle - \langle\tau\rangle\left(\varphi_\tau - \gamma\langle\alpha\rangle T_\tau\right)\delta_q \ ,$$

leading to the relationship

$$\theta = \varphi_\tau - \gamma\langle\alpha\rangle T_\tau$$

Using $\varphi_G = \langle\alpha\rangle\langle\tau\rangle\varphi_\tau$ and $T_G = -\langle\tau\rangle(1 - \langle\alpha\rangle T_\tau)$, we obtain

$$\lambda = \langle\alpha\rangle\langle\tau\rangle\varphi_\tau + \gamma\langle\alpha\rangle\langle\tau\rangle(1 - \langle\alpha\rangle T_\tau)$$

And therefore we obtain:

$$\frac{\lambda}{\langle\alpha\rangle\langle\tau\rangle} = \varphi_\tau + \gamma(1 - \langle\alpha\rangle T_\tau) = \varphi_\tau - \gamma\langle\alpha\rangle T_\tau + \gamma \ .$$

Hence, from the measurements of θ and λ, we obtain Equation 1 in the main text:

$$\theta\langle\alpha\rangle\langle\tau\rangle = \lambda - \gamma\langle\alpha\rangle\langle\tau\rangle \ . \tag{7}$$

Quite simply, Eq. (7) states that the overall correction to size over a cell cycle has to be the sum of a correction due to modulation of timing and a correction due to growth rate. The same approach can be readily generalized to a cell-cycle stage.

For the bacteria datasets, both $\langle\alpha\rangle$ and $\langle\tau\rangle$ are accessible, therefore this relationship can be directly tested. Another (equivalent) consistency test is discussed in the following section. For mammalian cells, since α cannot be measured directly, we use the approximation $\langle\alpha\rangle\langle\tau\rangle \approx \langle G\rangle$ (Fig. 7f). To verify that this approximation was correct, we repeated the analysis shown in Fig. 7e in bacteria with this approximate normalization, using $\langle G\rangle$ (Supplementary Fig. 8c). The results are very similar with the two normalizations.

4 Test of the balance equation on bacteria

Both λ and θ can be determined directly from the covariance between measured variables. Since, by definition

$$\langle G\rangle_q = \langle G\rangle - \lambda\delta_q$$

and

$$\mathrm{cov}(G,q) := \langle\langle G\rangle_q\delta_q\rangle \ ,$$

we have that

$$\mathrm{cov}(G,q) := -\lambda\sigma_q^2 \ ,$$

where σ_q^2 is the variance of q. Analogously, we have that

$$\mathrm{cov}(\tau,q) := -\langle\tau\rangle\theta\sigma_q^2 \ .$$

Using the above relationships, we can obtain λ and θ using

$$\lambda = -\frac{\mathrm{cov}(G,q)}{\sigma_q^2} \ ,$$

and:

$$\theta = -\frac{\mathrm{cov}(\tau,q)}{\langle\tau\rangle\sigma_q^2} \ .$$

Supplementary Fig. 8a shows a comparison of the values obtained for λ and θ from either the covariances or a linear fit testing the relationships (equations 5 and 6 respectively). The two methods are in excellent agreement for all data sets, except Raji and RPE1 cells (λ estimate) and HT29-hgem cells(θ estimate), where evident outliers are visible from the scatter plots (Supplementary Fig. 2a-c). In these cases, to compute the final Fig. 7f, we used the estimates from the linear fit on the bins which were more robust to noise than the covariance estimate.

Eq. (7) predicts a value of γ using the measured values of λ and θ

$$\gamma_{pred} = \frac{1}{\sigma_q^2 \langle\alpha\rangle\langle\tau\rangle} \left(-\mathrm{cov}(G,q) + \mathrm{cov}(\tau,q)\langle\alpha\rangle\right)$$

If individual growth rates are available, as with the bacteria datasets, γ can also be estimated directly as

$$\gamma_{meas} = -\frac{\mathrm{cov}(\alpha,q)}{\sigma_q^2\langle\alpha\rangle} \ .$$

Supplementary Fig. 8b shows that the measured value γ_{meas} and the predicted value γ_{pred} are in very good agreement, as expected from our theoretical considerations.

5 Non-exponential growth

As discussed above, for cells that do not grow exponentially the control parameter γ in Eq. (4) should not necessarily be interpreted as an active modulation of the growth rate. This section quantifies the predicted correlations in the case of sub-exponential growth.

5.1 Linear growth

We start revieweing the case of linear growth. If cells grow linearly, we have that

$$V(t) = V_0 + V_c g t \ ,$$

where g has dimension of the inverse of time and V_c dimension of size. At division we obtain

$$V_f = V_0 + V_c g \tau \ .$$

Therefore, for cells that grow linearly and divide according to a timer, for which $\theta = 0$ by definition, $V_f - V_0$ does not depend on V_0. Hence, they behave as an adder (i.e., $\lambda = 1/2$). Applying equation 7 to this case, one would obtain a value of γ different from zero, so that the control appears to operate fully through specific growth-rate modulation (which is correct, as there is no timing modulation for a timer). However, the size correction is not active, since there are also strong correlations between α and τ, since α is defined as $1/\tau \log(V_f/V_0)$ and linear growth ($V_f = V_0 + V_c g\tau$) makes it a function of τ. In other words, in this case one has to be careful not to mistake the intrinsic properties of a timer for a linearly growing cell with an active mechanism of growth modulation.

5.2 Polynomial growth

We now derive the predicted correlation pattern in the case of more general sub-exponential growth, and in particular for cells whose volume follows the equation

$$\frac{dV}{dt} = gV^{1-\beta}V_c^{\beta} \ . \tag{8}$$

This model reduces to linear growth in the case of $\beta = 1$ and to exponential growth in the case $\beta = 0$, for intermediate cases, growth is polynomial. This growth model depends only on the

parameter β and another one effective parameter gV_c^β. We separate the contribution of g (with dimension of the inverse of time) and V_c (with dimension of size). We will show that $\gamma > 0$ (size-restoring growth rate modulation-based homeostasis) emerges in the case of $\beta > 0$. Hence, sublinear growth may carry some homeostatic control on size.

Defining $q := \log(V/V_c)$, one obtains

$$\frac{dq}{dt} = ge^{\beta q} ,$$

whose solution is

$$q(t) = \frac{1}{\beta} \log\left(e^{\beta q_0} + \beta g t\right) .$$

We have therefore that final logarithmic size q_f of a cell with initial size q_0 and division time τ is equal to

$$q_f = \frac{1}{\beta} \log\left(e^{\beta q_0} + \beta g \tau\right) .$$

As above, we define $G = q_f - q_0$ and, the exponential growth rate α as G/τ. In the case of $\beta = 0$, $g = \alpha$. Our goal is to show that, for $\beta > 0$ a homeostatic control on α emerges, i.e.,

$$\alpha := \frac{G}{\tau} = \frac{q_f - q_0}{\tau} = \langle\alpha\rangle(1 - \gamma\delta_q) ,$$

with $\gamma \neq 0$ for $\beta > 0$.

Here we assume no direct control on g (i.e., it is a constant independent of the initial size) and no dependence of τ on δ_α ($T_\tau = 0$). We have therefore

$$\tau = \langle\tau\rangle\left(1 - \varphi_\tau\delta_q\right) + \nu_\tau .$$

Using the definition of $G = q_f - q_0$ and $q_0 = \langle q_0\rangle + \delta_q$, we have

$$G = q_f - q_0 = \frac{1}{\beta} \log\left(e^{\beta\langle q_0\rangle + \beta\delta_q} + \beta g\langle\tau\rangle(1 - \varphi_\tau\delta_q) + g\nu_\tau\right) - \langle q_0\rangle - \delta_q ,$$

which, by expanding around $\delta_q = 0$, simplifies to

$$G = \frac{1}{\beta} \log\left(e^{\beta\langle q_0\rangle} + \beta g\langle\tau\rangle + \beta(1 - g\langle\tau\rangle\varphi_\tau)\delta_q + g\nu_\tau\right) - \langle q_0\rangle - \delta_q ,$$

and then

$$G = \frac{1}{\beta} \log\left(e^{\beta\langle q_0\rangle} + \beta g\langle\tau\rangle\right) - \langle q_0\rangle + \left(\frac{1 - g\langle\tau\rangle\varphi_\tau}{e^{\beta\langle q_0\rangle} + \beta g\langle\tau\rangle} - 1\right)\delta_q + \text{noise} .$$

Since the single-cell growth dynamics is specified by the parameter $gV_c^{1-\beta}$, we are free to choose V_c arbitrarily. V_c enters in the definition of $q_0 = \log(V_0/V_c)$ and sets the value of $\langle q_0\rangle$. The simplest choice is to consider $\langle q_0\rangle = 0$. Note that this can be done in full generality, as it only affects the definition of g in the case $\beta > 0$. We obtain therefore

$$G = \frac{1}{\beta} \log\left(1 + \beta g\langle\tau\rangle\right) + \left(\frac{1 - g\langle\tau\rangle\varphi_\tau}{1 + \beta g\langle\tau\rangle} - 1\right)\delta_q + \text{noise} =: \langle G\rangle - \varphi_G\delta_q + \nu_G .$$

We obtain then

$$\langle G\rangle = \frac{1}{\beta} \log\left(1 + \beta g\langle\tau\rangle\right) .$$

Assuming stationarity, we have that $\langle G\rangle = \log 2$, and therefore

$$g\langle\tau\rangle = \frac{2^\beta - 1}{\beta} ,$$

from which we obtain

$$G = \langle G\rangle - \left(\frac{1-2^{-\beta}}{\beta}\varphi_\tau + 1 - 2^{-\beta}\right)\delta_q + \text{noise} .$$

In the case $\beta = 0$ we obtain $\varphi_G = \varphi_\tau \log 2$. Note that if $\beta = 0$, the factor $\log 2$ is equal to $g\langle\tau\rangle = \langle\alpha\rangle\langle\tau\rangle$ and we recover $\varphi_G = \langle\alpha\rangle\langle\tau\rangle\varphi_\tau$, obtained for exponential growth. For linear growth ($\beta = 1$) we have instead $\varphi_G = (\varphi_\tau + 1)/2$. As expected, a timer ($\varphi_\tau = 0$) corresponds to an adder ($\varphi_G = \lambda = 0.5$) for linear growth. This expresses the well-known fact that, for linear growth, a timer coincides with an adder and is able to perform size correction[26], see also section 5.1

The exponential growth rate is defined as

$$\alpha = \frac{G}{\tau} .$$

By expanding for small δ_q, we obtain an equation giving the fluctuation pattern of α,

$$\alpha = \langle\alpha\rangle(1-\gamma\delta_q) + \text{noise} = \frac{\langle G\rangle - \varphi_G\delta_q + \nu_G}{\langle\tau\rangle(1-\varphi_\tau\delta_q) + \nu_\tau} = \frac{\langle G\rangle}{\langle\tau\rangle}(1+\varphi_\tau\delta_q) - \frac{\varphi_G}{\langle\tau\rangle}\delta_q + \text{subleading terms} .$$

By imposing stationarity ($\langle G\rangle = \log 2$) and neglecting subleading terms, we finally obtain

$$\gamma = \frac{\varphi_G}{\langle\alpha\rangle\langle\tau\rangle} - \varphi_\tau = \left(\frac{1-2^{-\beta}}{\beta\langle\alpha\rangle\langle\tau\rangle} - 1\right)\varphi_\tau + \frac{1-2^{-\beta}}{\langle\alpha\rangle\langle\tau\rangle} , \tag{9}$$

which is equal to zero if $\beta = 0$ (exponential growth, for which $\langle\alpha\rangle\langle\tau\rangle = \log 2$) and always positive if $\beta > 0$. The limit case $\varphi_\tau = 0$, $\beta = 1$ (timer, linear growth) corresponds to an adder ($\lambda = 1/2$) where all the control is exerted by the growth mode ($\gamma\langle\alpha\rangle\langle\tau\rangle = 1/2$). Note that a positive γ corresponds to homeostatic correction (larger cells grow slower). For intermediate values of β, Eq. (9) gives the expected contribution of the growth mode to size correction, which should be disentangled from size-based growth rate changes at key checkpoints. In order to distinguish such an active modulation of the growth rate from passive correction due to sublinear growth (a subtle question), one needs to access many high-resolution time tracks of growing cells, beyond the currently available statistics.

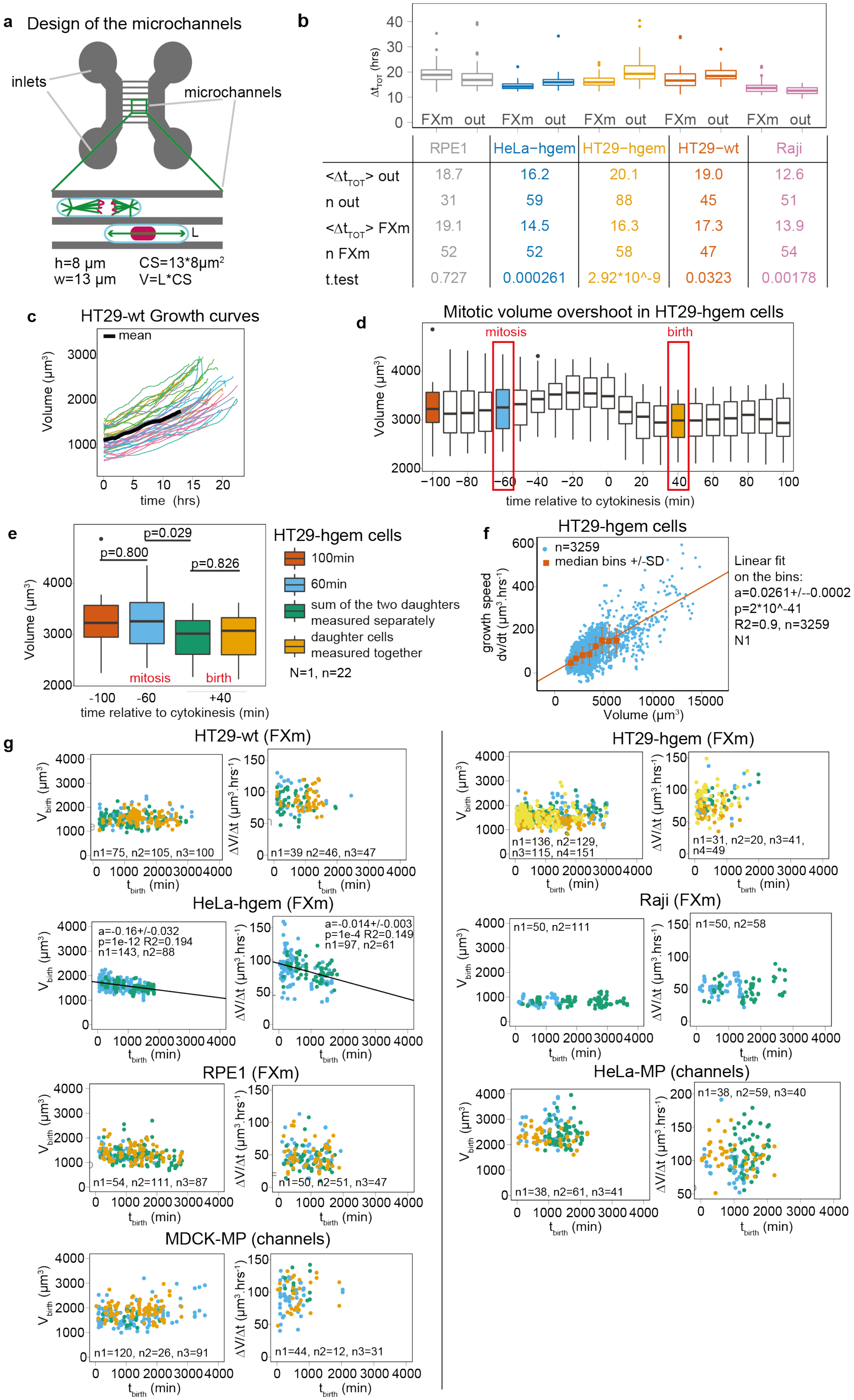
a
Design of the microchannels
inlets
microchannels
L
h=8 µm
w=13 µm
CS=13*8µm²
V=L*CS
b
Δt_TOT (hrs)
FXm out FXm out FXm out FXm out FXm out
RPE1 HeLa−hgem HT29−hgem HT29−wt Raji
<Δt_TOT> out 18.7 16.2 20.1 19.0 12.6
n out 31 59 88 45 51
<Δt_TOT> FXm 19.1 14.5 16.3 17.3 13.9
n FXm 52 52 58 47 54
t.test 0.727 0.000261 2.92*10^-9 0.0323 0.00178
c
HT29-wt Growth curves
mean
Volume (µm³)
time (hrs)
d
Mitotic volume overshoot in HT29-hgem cells
mitosis
birth
Volume (µm³)
time relative to cytokinesis (min)
e
p=0.029
p=0.800
p=0.826
mitosis
birth
Volume (µm³)
time relative to cytokinesis (min)
HT29-hgem cells
100min
60min
sum of the two daughters measured separately
daughter cells measured together
N=1, n=22
f
HT29-hgem cells
n=3259
median bins +/-SD
growth speed dv/dt (µm³.hrs⁻¹)
Volume (µm³)
Linear fit on the bins:
a=0.0261+/--0.0002
p=2*10^-41
R2=0.9, n=3259
N1
g
HT29-wt (FXm)
n1=75, n2=105, n3=100
n1=39 n2=46, n3=47
HT29-hgem (FXm)
n1=136, n2=129, n3=115, n4=151
n1=31, n2=20, n3=41, n4=49
HeLa-hgem (FXm)
a=-0.16+/-0.032
p=1e-12 R2=0.194
n1=143, n2=88
a=-0.014+/-0.003
p=1e-4 R2=0.149
n1=97, n2=61
Raji (FXm)
n1=50, n2=111
n1=50, n2=58
RPE1 (FXm)
n1=54, n2=111, n3=87
n1=50, n2=51, n3=47
HeLa-MP (channels)
n1=38, n2=61, n3=41
n1=38, n2=59, n3=40
MDCK-MP (channels)
n1=120, n2=26, n3=91
n1=44, n2=12, n3=31
V_birth (µm³)
ΔV/Δt (µm³.hrs⁻¹)
t_birth (min)

Supplementary Figure 1, related to Figure 1: Controls for measurement of volume in FXm and microchannels

a, Design of the microchannels (h: height, w: width, L: cell length, V: cell volume, CS: cross section). Scheme: blue: cell membrane, magenta: DNA, green: microtubules, grey bars: microchannels).

b, Top: Boxplot showing the duration of cell cycle length (Δt_{TOT}) inside ('FXm') and outside ('out') the FXm measurement device. Bottom: table with the average cell cycle duration ($\langle \Delta t_{TOT} \rangle$) and the p-value of a Welch's t test comparing the mean cell cycle duration in both conditions for each cell line.

c, Examples of complete single cell growth curves obtained with the FXm method (n=45, N=2). The curves start at birth and end at mitosis. Black curve shows the average of all the curves from birth to the time of mitosis of the shortest cell cycle.

d, Boxplot of the volumes measured at different time-points relative to cytokinesis in HT29-hgem cells (n=22, N=1). The colored time-points correspond to the time-points 100 minutes before (red), 60 minutes before (blue, time-point where volume at mitosis was measured) and 40 minutes after (orange, time-point where volume at birth was measured) cytokinesis. These time-points are compared in Supplementary Fig. 1e.

e, Average volume measured 100 mins before, 60 minutes before and 40 minutes after cytokinesis. p-values are p-values from a pairwise t test comparing the means.

f, Instantaneous growth speed (dv/dt) as a function of volume of individual or groups of cells. Each blue point is the instantaneous growth speed over a small portion of a volume growth curve (see Supplementary Fig. 7a).

g, Plots of volume at birth (V_{birth}) vs. time at birth (t_{birth}) and average growth speed ($\Delta V/\Delta t$) vs. t_{birth} in the experiment (time = 0min corresponds to the beginning of image acquisition) for all experiments shown in Fig. 2 and 4. The colors correspond to independent replicates of the experiment, the legend indicates the number of events in each replicate (i.e. n1 for number in experiment 1, etc…). The results of a robust linear fit and the corresponding slope are shown when the coefficient of determination (R2) is above 0.1.

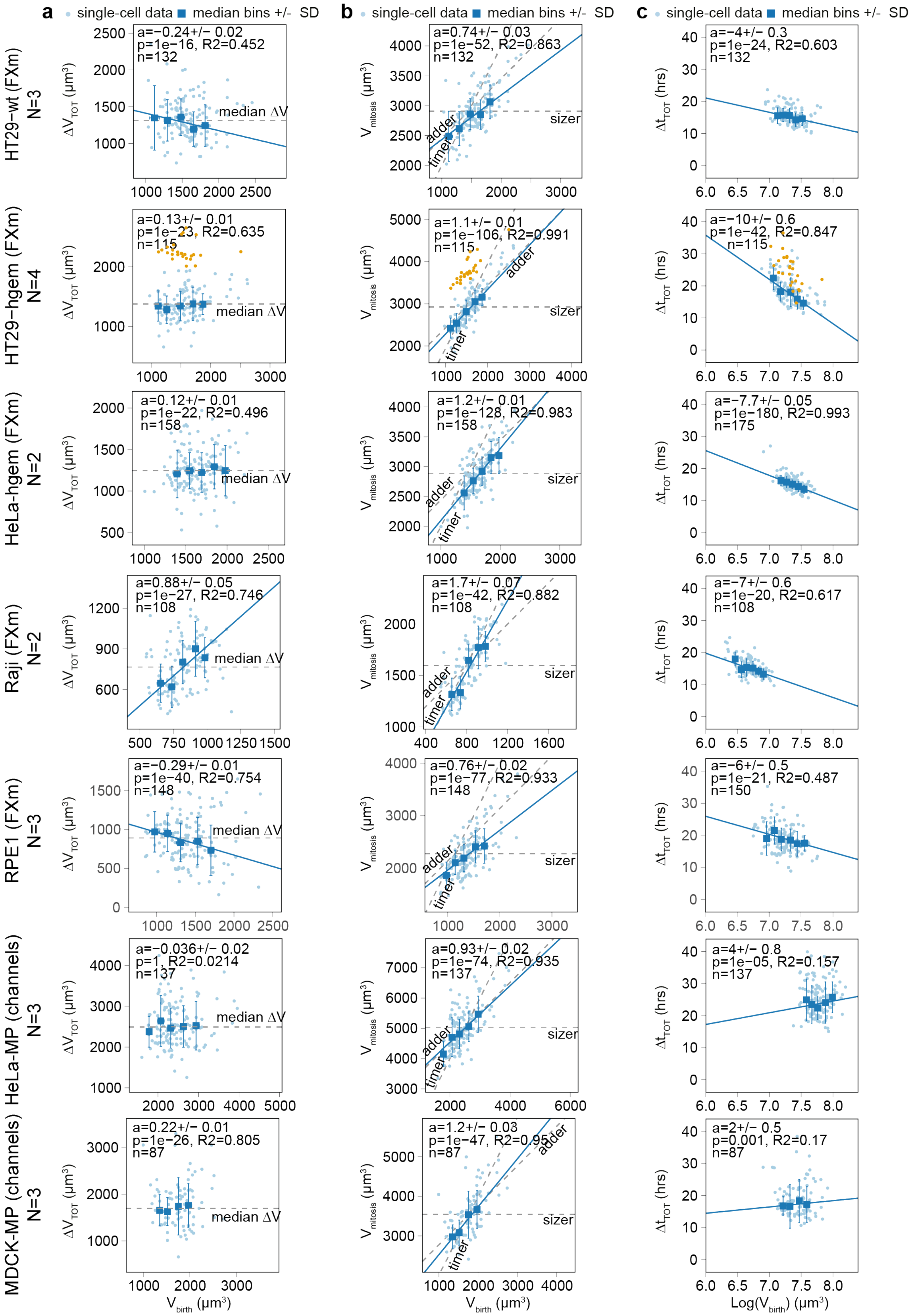

d

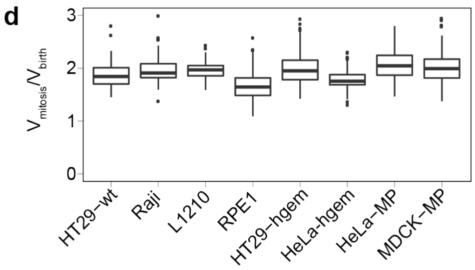

cell line	$\langle V_{mitosis}/V_{birth} \rangle$	n	N
HT29-wt	1.9	132	3
Raji	2.0	108	2
L1210	2.0	121	
RPE1	1.7	148	2
HT29-hgem	2.0	141	4
HeLa-hgem	1.8	158	2
HeLa-MP	2.1	137	3
MDCK-MP	2.1	87	3

Supplementary Figure 2, related to Figure 2: General homeostatic behavior in cultured mammalian cells

a-c Plots for each individual cell type shown in Fig. 2b measured either with the FXm or with the microchannels devices. **a** Added volume in the cell cycle (ΔV_{tot}) as a function of volume at mitosis ($V_{mitosis}$). The dashed line indicates the median added volume during the cell cycle. **b** $V_{mitosis}$ vs. volume at birth (V_{birth}). The dashed lines indicate the predicted slopes for timer (assuming exponential growth, slope = 2, intercept = 0), adder (slope = 1, intercept = $\langle \Delta V_{tot} \rangle$) and sizer (slope = 0, intercept = $\langle V_{mitosis} \rangle$. **c** Total cell cycle duration (ΔT_{tot}) vs. volume at birth (V_{birth}). Solid blue line indicates a linear fit performed on the median bins weighted by the number of observations in each bin.

For the HT29-hgem cells, a small population of cells gained more volume than the average (more than 2000µm3, orange points, n=26, 18% of the cells). A possible explanation is that these cells represent a sub-clone (from this polyclonal population stably expressing hgeminin-mCherry) which had a longer G1 (mean G1 duration, subpopulation: m=13.2, mean G1 duration, other cells: m=8.7, Welch t.test comparing the means: p=9.5*10^-5; mean S-G2 duration, subpopulation: m=11.5, mean S-G2 duration, other cells: m=10.1, Welch t test comparing the means: p=3.2*10^-5). The results of the statistical analysis performed on the population after removal of the subpopulation (n=115) is indicated on the graphs. The results of the statistical analysis performed on the whole populations (n=141) yield similar results: for ΔV_{tot} vs. V_{birth}, P=0.13, n.s.; for $V_{mitosis}$ vs V_{birth}, a=1.2 ***, R2=0.956; for ΔT_{tot} vs. V_{birth}, P= -0.388 ***. (p>0.5 n.s., p<0.01:**, p<0.001: ***).

d Left: boxplot of overall replicative growth (volume at mitosis divided by volume at birth) for all the cell types analyzed in Fig. 2b. Right: table summarizing the values in the boxplot. HeLa-hgem and RPE1 cells are on average lower than the rest of the datasets. For HeLa-hgem, this is explained by the fact that the fluorescent probe used for FXm is slightly accumulated in the cell, which induces a small but significant decrease in volume (see Supplementary Fig. 1g). For RPE1 cells, as there was no visible accumulation of the fluorescent probe in the cells, the reason for the lower overall replicative growth is unclear. In Fig. 2b, the bins for RPE1 and HeLa-hgem are shifted to the right compared with the other datasets because $\langle V_{birth} \rangle / \langle V_{mitosis} \rangle$ is higher for these datasets.

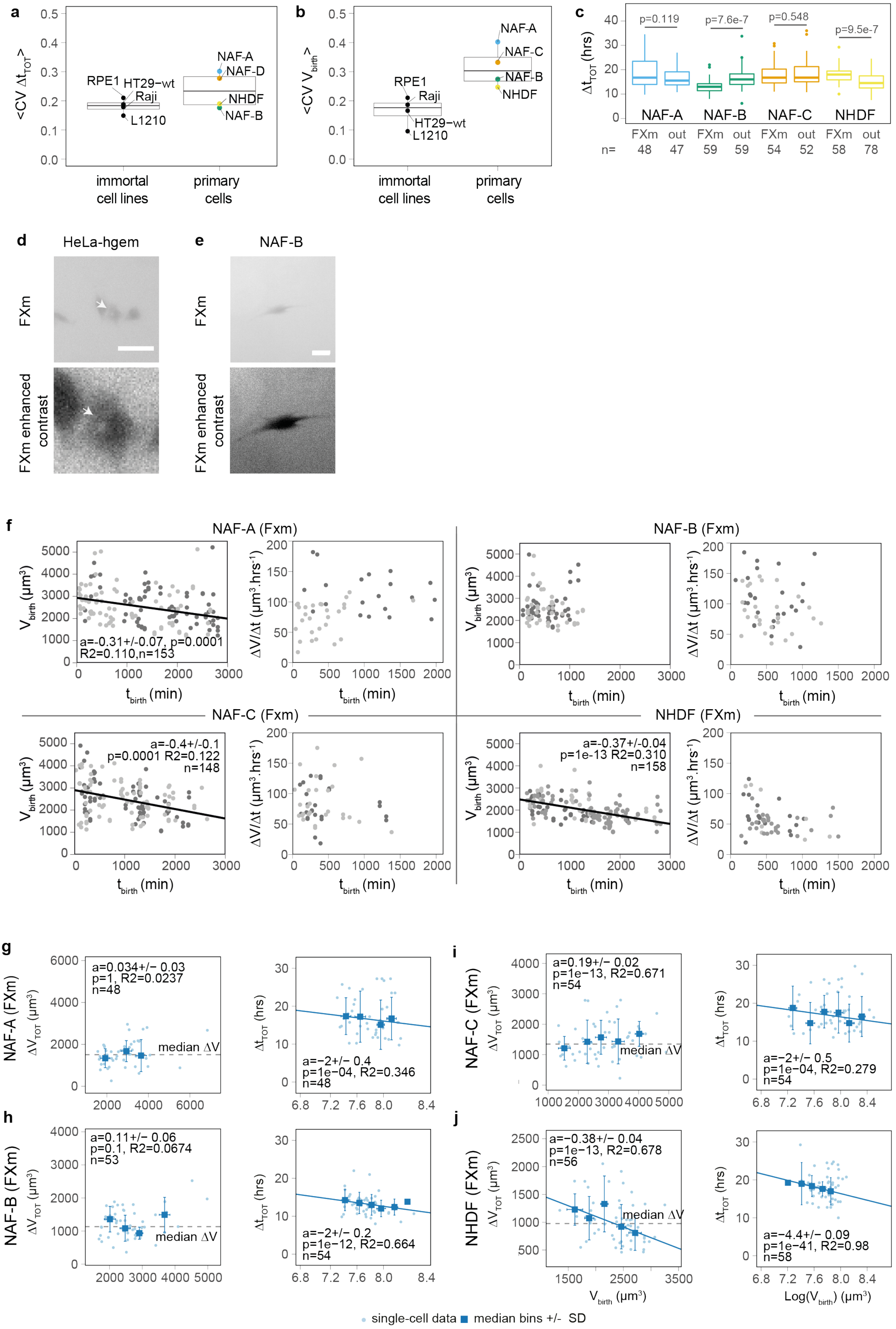
a
b
c
<CV Δt_TOT>
<CV V_birth>
RPE1
HT29-wt
Raji
L1210
NAF-A
NAF-D
NHDF
NAF-B
NAF-C
immortal cell lines
primary cells
p=0.119
p=7.6e-7
p=0.548
p=9.5e-7
Δt_TOT (hrs)
FXm out FXm out FXm out FXm out
n= 48 47 59 59 54 52 58 78
d
e
HeLa-hgem
NAF-B
FXm
FXm enhanced contrast
f
NAF-A (Fxm)
NAF-B (Fxm)
NAF-C (Fxm)
NHDF (FXm)
V_birth (µm³)
ΔV/Δt (µm³.hrs⁻¹)
t_birth (min)
a=-0.31+/-0.07, p=0.0001
R2=0.110,n=153
a=-0.4+/-0.1
p=0.0001 R2=0.122
n=148
a=-0.37+/-0.04
p=1e-13 R2=0.310
n=158
g
h
i
j
NAF-A (FXm)
NAF-B (FXm)
NAF-C (FXm)
NHDF (FXm)
ΔV_TOT (µm³)
Δt_TOT (hrs)
a=0.034+/− 0.03
p=1, R2=0.0237
n=48
median ΔV
a=−2+/− 0.4
p=1e−04, R2=0.346
n=48
a=0.11+/− 0.06
p=0.1, R2=0.0674
n=53
a=−2+/− 0.2
p=1e−12, R2=0.664
n=54
a=0.19+/− 0.02
p=1e−13, R2=0.671
n=54
a=−2+/− 0.5
p=1e−04, R2=0.279
n=54
a=−0.38+/− 0.04
p=1e−13, R2=0.678
n=56
a=−4.4+/− 0.09
p=1e−41, R2=0.98
n=58
V_birth (µm³)
Log(V_birth) (µm³)
single-cell data
median bins +/- SD

Supplementary Figure 3, related to Figure 3: Controls for the experiments on primary cells

a-b Boxplot representing the average coefficient of variation (CV) of cell cycle duration (Δt_{TOT}) **(a)** and volume at birth (V_{birth}) **(b)** for immortal cell lines (cancerous wild-type HT29, Raji and L1210, immortalized wild-type RPE1) and primary cells. Each point is the average of the coefficients of variation calculated in independent replicates for each cell type (RPE1, N=3; HT29-wt, N=3, Raji, N=2, L1210 are data from Son and colleagues[22]; NAF-A, N=2; NAF-B, N=2; NAF-C, N=2; NHDF, N=3).

c Boxplot showing the duration of cell cycle length inside ('FXm') and outside ('out') the FXm measurement device for the three samples of NAFs and NHDF cells. N, the number of independent replicates is: NAF-A, N=2; NAF-B, N=2; NAF-C, N=2; NHDF, N=3.

d-e Example of one HeLa-hgem cell showing an intracellular accumulation of the fluorescent probe (white arrow) **(d)** and one NAF-B showing no dextran accumulation **(e)** during an acquisition for FXm measurement. Bottom pictures are magnifications of the upper pictures with enhanced contrast to highlight potential intracellular fluorescence signal. Scale bar is 50µm.

f Controls for the quality of growth for all the experiments on primary cells showing V_{birth} vs. t_{birth} in the experiment and average growth speed ($\Delta V/\Delta t$) vs. t_{birth} in the experiment. (time = 0min corresponds to the beginning of the image acquisition). The results of a robust linear fit and the corresponding slope are shown when the coefficient of determination (R2) is above 0.1. Grey levels correspond to independent replicates (NAF-A: n=20, n=28 ; NAF-B, n=27, n=26 ; NAF-C; n=25, n=29 ; NHDF: n=19, n=10, n=27).

g-j Analysis of the homeostatic behaviour of primary NAFs and NHDF. Left: Added volume in the cell cycle (ΔV_{tot}) as a function of volume at birth. The dashed line indicates the median added volume during the cell cycle. Right: Total cell cycle duration vs. logarithm of volume at birth. For all the plots, the solid blue line indicates a linear fit performed on the median bins weighted by the number of observations in each bin.

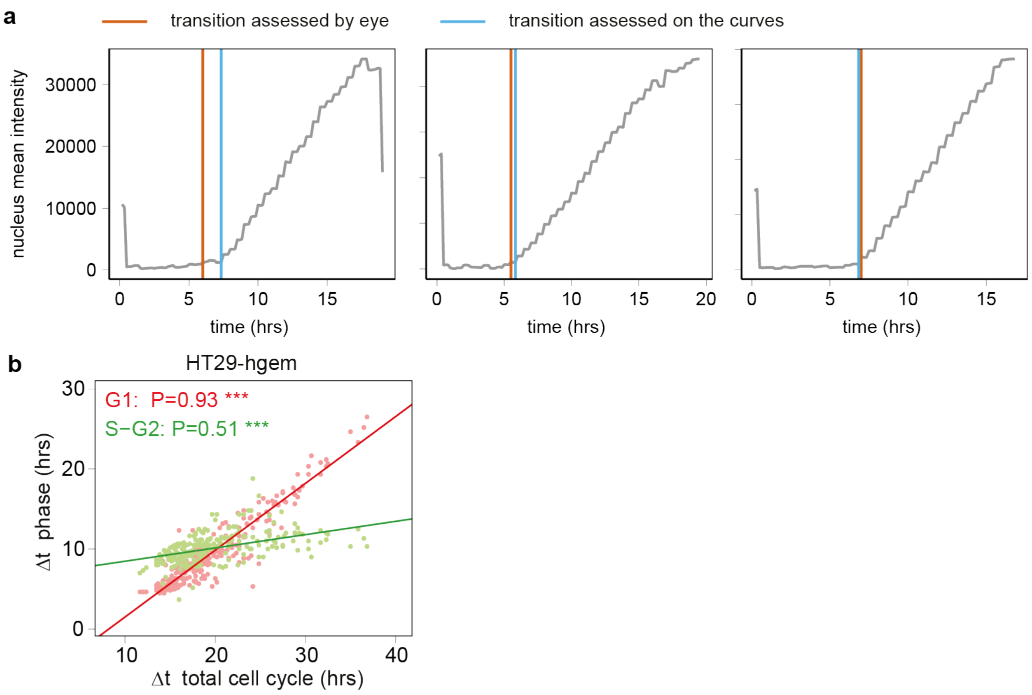

Supplementary Figure 4, related to Figure 4: Characterization of G1 and S-G2 in HT29

a, 3 examples of fluorescence intensity profile of hgeminin-mcherry in HT29 cells over time (from cell birth to cell mitosis). Comparison of the identification of the G1/S transition visually (red line) or from the slope transition on the fluorescence intensity curves (blue).

b, Duration of G1 or S-G2 phases (Δt phase) with respect to total cell cycle duration (Δt total cell cycle) in HT29 cells expressing hgeminin-mcherry (n=228, N=4). Pearson's correlation coefficient P and p-values ($p<0.001$: ***) are indicated for both phases. G1 duration correlates strongly with total cell cycle duration while S-G2 duration does less. (Lines represent robust linear fits on the points).

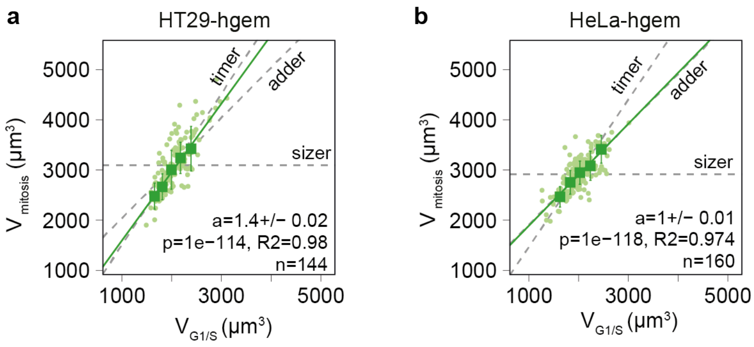

Supplementary Figure 5, related to Figure 5: Effective homeostatic behavior in S-G2 for HeLa and HT29

a-b, Volume at mitosis ($V_{mitosis}$) vs. volume at G1/S ($V_{G1/S}$) for HT29-hgem (N=4) **(a)** and HeLa-hgem (N=2) **(b)**. The dashed line indicate the predicted slopes for timer (assuming exponential growth, slope = $\langle V_{mitosis}/V_{G1/S}\rangle$, intercept = 0), adder (slope = 1, intercept = $\langle \Delta V_{S-G2}\rangle$) and sizer (slope = 0, intercept = $\langle V_{mitosis}\rangle$). Solid lines represent linear fits on the bins (green squares) weighted by the number of observations in each bin (dots). Error bars represent the s.d..

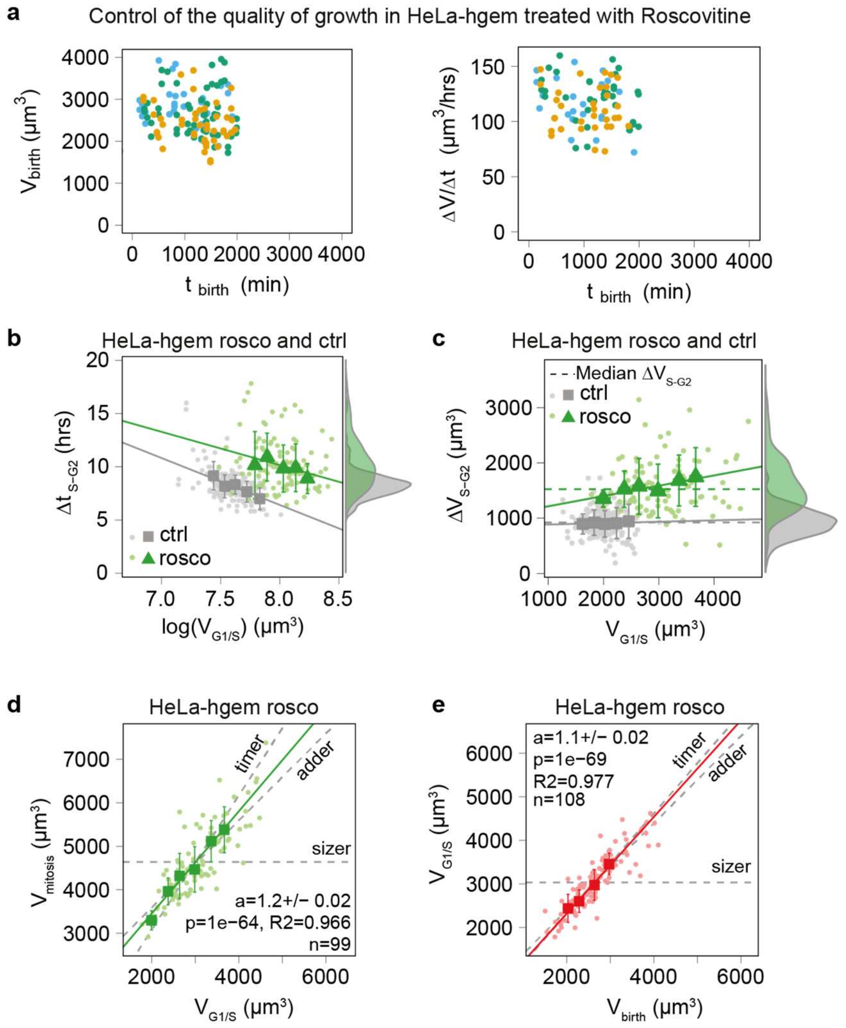

Supplementary Figure 6, related to Figure 6: Homeostatic behavior in Roscovitine treated HeLa cells

a, Control of the quality of growth for experiments with Roscovitine-treated HeLa-hgem cells ('rosco') : plots of volume at birth (V_{birth}) and average growth speed $\Delta V / \Delta t$ as a function of time at birth (t_{birth}) in the experiment (time = 0 min corresponds to the beginning of the image acquisition). The colors correspond to the 3 independent replicates of the experiment. N=3; V_{birth} vs. t_{birth}: n=35, n=56, n=45; $\Delta V/\Delta t$ vs. t_{birth}: n=27, n=47, n=33.

b, S-G2 duration (Δt_{S-G2}) as a function of the logarithm of volume at G1/S ($V_{G1/S}$) transition. Results from the linear fit (solid lines): ctrl: a=-4+/-0.2 p=1e-55, R2=0.790, n=172, N=2; rosco: a=-3 +/-0.3, p=2e-25, R2=0.561, n=107, N=3. Right distributions show kernel estimates for S-G2 duration distributions. S-G2 is on average longer for Roscovitine treated cells (ctrl: $\langle \Delta t_{S-G2} \rangle = 8$ hrs.; rosco: $\langle \Delta t_{S-G2} \rangle = 10$ hrs.; Welch t test comparing the mean: p=3*10^-14).

c, Added volume in S-G2 (ΔV_{S-G2}) as a function of $V_{G1/S}$. Results from the linear fit (solid lines): ctrl: a=0.025+/-0.005, p=1e-05, R2=0.149, n=160, N=2; rosco: a=0.2+/-0.01, p=1e-23, R2=0.701, n= 99, N=3). Right distributions show kernel estimates for ΔV_{S-G2} distributions. The median volume (dashed lines) is on average longer for Roscovitine treated cells (ctrl: $\langle \Delta V_{S-G2} \rangle = 900$ µm3; rosco: $\langle \Delta V_{S-G2} \rangle = 1600$ µm3; Welch t test comparing the mean: p=2*10^-16).

(continued next page)

Supplementary Figure 6 (continued)

d-e, Volume at mitosis ($V_{mitosis}$) vs. $V_{G1/S}$) **(d)** and $V_{G1/S}$ vs. V_{birth} **(e)** for HeLa-hgem treated with Roscovitine (N=3). Grey dashed lines indicate the predicted trends in the case of a timer (assuming exponential growth, slope = $\langle V_{mitosis}/V_{G1/S} \rangle$ or $\langle V_{G1/S}/V_{birth} \rangle$, respectively, intercept = 0), adder (slope = 1, intercept = $\langle \Delta V_{S-G2} \rangle$ or $\langle \Delta V_{G1} \rangle$ respectively) and sizer (slope = 0, intercept = $\langle V_{mitosis} \rangle$ or $\langle V_{G1/S} \rangle$ respectively). Results from the fit (solid lines) are indicated in the legend.

For plots in **b-e,** solid lines are linear fits on the median bins weighted by the number of observation in each bin. Squares or triangles represent median bins, error bars are s.d. and dots are single-cell observations.

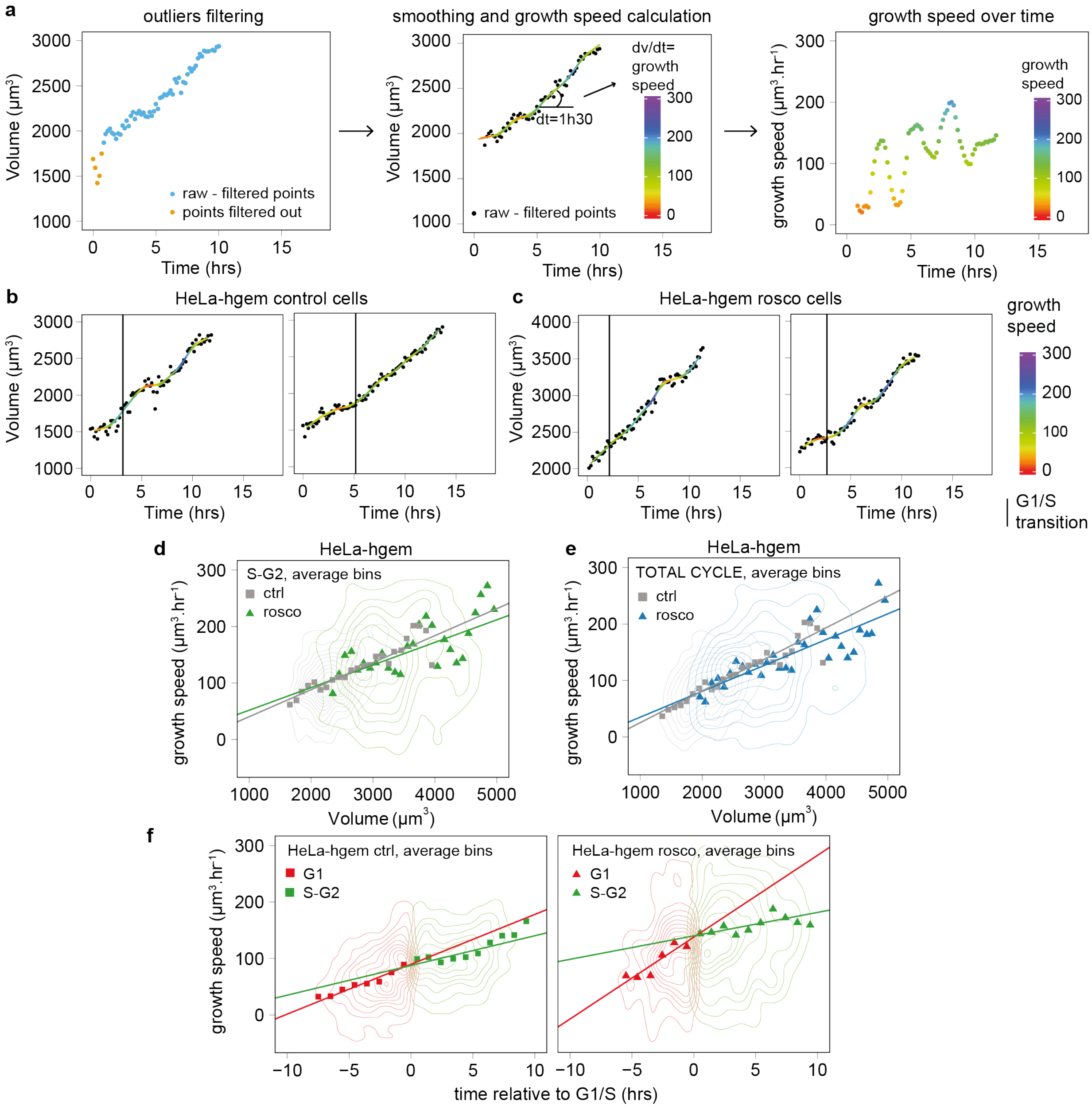

Supplementary Figure 7, related to Figure 6: Growth rate analysis on complete single cell volume curves

a, Example showing how single-cell volume curves were smoothed and how growth speed was calculated for one control HeLa-hgem cell. Left: outliers (orange points) corresponding to errors in the automated segmentation of the cell were removed from the data (blue points) (see Methods). Middle: calculation of the instantaneous growth speed. The dark points are the data kept after filtering out the outliers. Colored lines represent local robust linear fits performed to estimate the instantaneous growth speed and are colored as a function of the slope of this fit to indicate the local value of growth speed (scale bar). Right: growth speed as a function of time. Points are colored as a function of growth speed (scale-bar).

b-c, Examples of complete single-cell volume curves for 2 HeLa-hgem control cells (**b**) and 2 Roscovitine treated cells (**c**). Dark points represent the raw measurement and the vertical line the time of G1/S transition. Colored lines represent local robust linear fits performed to estimate the instantaneous growth speed and are colored as a function of the slope of this fit to indicate the local value of growth speed (scale bar).

(continued next page)

Supplementary Figure 7 (continued)

d-e, Instantaneous growth speed during S-G2 **(d)** or the whole cell cycle **(e)** as a function of volume, with bivariate kernel densities (concentric circles) and average bins (squares and triangles) for control HeLa-hgem cells ('ctrl', n=119, N=1) and Roscovitine treated HeLa-hgem cells ('rosco', n=49, N=2). Lines show linear regressions on average bins weighted by the number of event in each bin; S-G2, ctrl: a=0.0478+/-0.0003, $p \approx 0$, R2=0.86; S-G2, rosco: a=0.0400+/-0.0010, $p \approx 0$, R2=0.49; total cycle, ctrl: a=0.0559+/-0.000, $p \approx 0$, R2=0.92; total cycle, rosco: a=0.0457+/-0.0006, $p \approx 0$, R2=0.69.

f, Instantaneous growth speed during the cell cycle as a function of time from G1/S transition, with bivariate kernel densities and average bins for control (left, n=119) and Roscovitine treated (right, n=49) HeLa-hgem cells. Red and green lines show linear regression on average bins weighted by the number of event in each bin for G1 and S-G2 phases respectively; ctrl, G1: a=8.83+/-0.05, $p \approx 0$, R2=0.92; ctrl, S-G2: a=5.26+/-0.05, $p \approx 0$, R2=0.66; rosco, G1: a=14.5+/-0.3, $p \approx 0$, R2=0.77; rosco, S-G2: a=4.15+/-0.09, $p \approx 0$, R2=0.55.

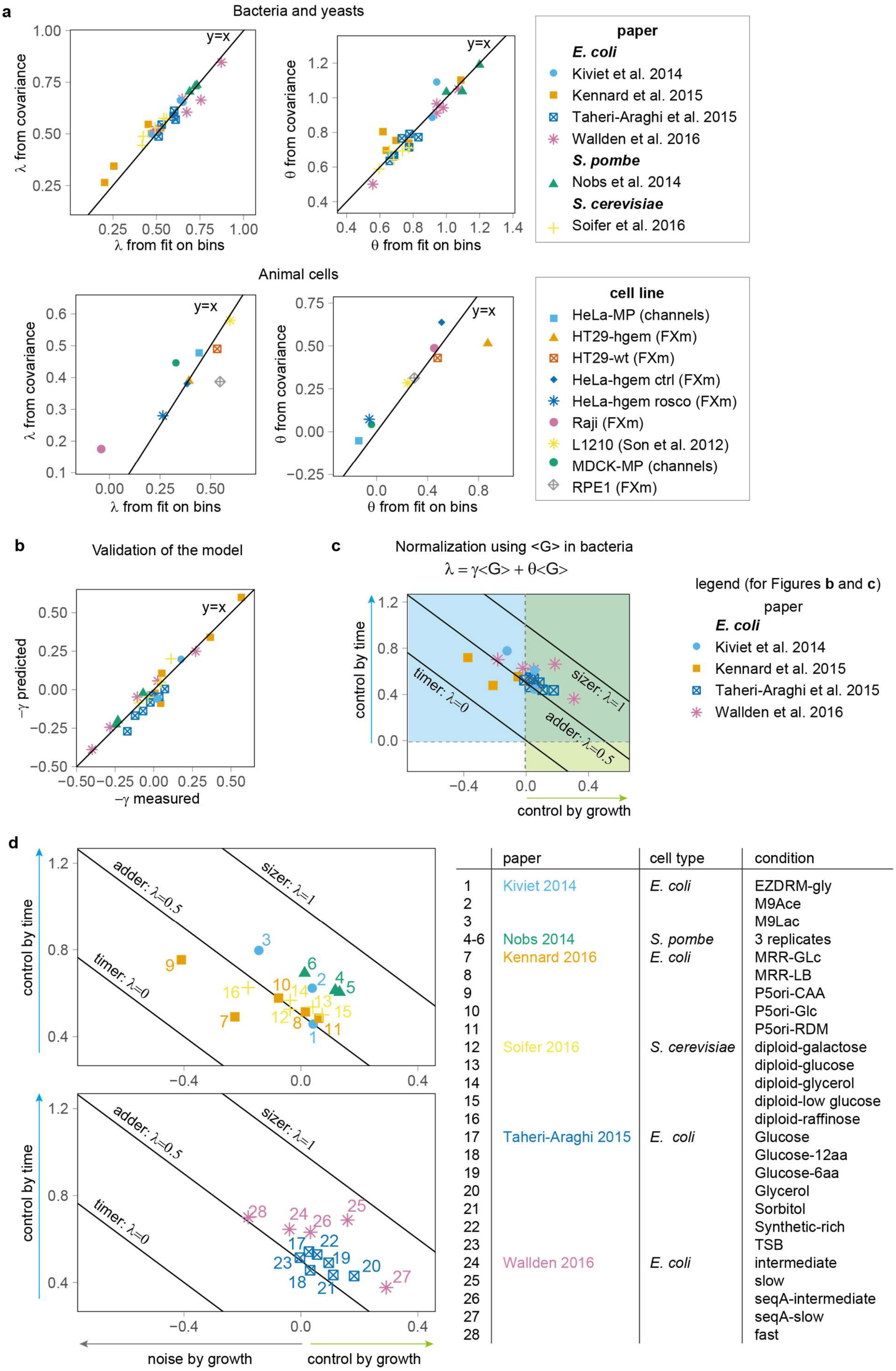

Supplementary Figure 8: Validation of the mathematical framework

Supplementary Figure 8, related to Figure 7: Validation of the mathematical framework

a, Comparison of the estimation of the values of λ and θ using a calculation of the covariance or a linear fit on the binned data (see Supplementary Note 1 and Fig. 7a). Each point corresponds to one dataset. Top: datasets from bacteria and yeasts studies[18,27–31], bottom: datasets from our experiments and obtained on L1210 (kindly sent by Son and colleagues, ref.[22]). The line represents the relation y=x. In Fig. 7f, the values of θ for HT29-hgem and λ for RPE1 are those calculated from the linear fit on the bins since the covariance estimates were more sensitive to the noise in these datasets.

b, Validation of the model using bacteria: comparison of γ as predicted by the model (see Supplementary Note 1) with γ calculated from a fit on the bins (as in Fig. 7c). Each point corresponds to one dataset. The line represents the relation y=x.

c, Same results as in Fig. 7e for bacteria datasets but with the normalization of γ and θ with average doubling $\langle G \rangle$ instead of the product of average growth rate $\langle \alpha \rangle$ and average cell cycle duration $\langle \tau \rangle$ as was used for mammalian cells and yeasts results (where the average growth rate $\langle \alpha \rangle$ is not available) (see Supplementary Note 1). Each point corresponds to one dataset.

d, Results as in Fig. 7e with the detail of the growth condition for each dataset analyzed for yeasts and bacteria. For more precisions about the experimental conditions, please refer to the corresponding publications[18,27,28,30–32].

Supplementary References

1. Osella, M., Tans, S. J. & Cosentino Lagomarsino, M. Step by Step, Cell by Cell: Quantification of the Bacterial Cell Cycle. *Trends Microbiol.* **25,** 250–256 (2017).
2. Facchetti, G., Chang, F. & Howard, M. Controlling cell size through sizer mechanisms. *Curr. Opin. Syst. Biol.* 1–7 (2017). doi:10.1016/j.coisb.2017.08.010
3. Jorgensen, P. & Tyers, M. How cells coordinate growth and division. *Curr. Biol.* **14,** R1014-27 (2004).
4. Mitchison, J. M. Growth During the Cell Cycle. *Int. Rev. Cytol.* **226,** 165–258 (2003).
5. Turner, J. J., Ewald, J. C. & Skotheim, J. M. Cell size control in yeast. *Curr. Biol.* **22,** (2012).
6. Jun, S. & Taheri-araghi, S. Cell-size maintenance: Universal strategy revealed. *Trends Microbiol.* **23,** 4–6 (2015).
7. Killander, D. & Zetterberg, A. quantitative cytochemical studies on interphase growth II derivation of synthesis curves from the distribution of DNA, RNA and mass values of individual mouse fibroblasts in vitro. *Exp. Cell Res.* **39,** 22–32 (1965).
8. Dolznig, H., Grebien, F., Sauer, T., Beug, H. & Müllner, E. W. Evidence for a size-sensing mechanism in animal cells. *Nat. Cell Biol.* **6,** 899–905 (2004).
9. Liu, S. *et al.* Size uniformity of animal cells is actively maintained by a p38 MAPK-dependent regulation of G1-length. *Elife* **7,** e26947 (2018).
10. Varsano, G. *et al.* Probing Mammalian Cell Size Homeostasis by Article Probing Mammalian Cell Size Homeostasis by Channel-Assisted Cell Reshaping. *CellReports* **20,** 397–410 (2017).
11. Goranov, A. I. & Amon, A. Growth and division-not a one-way road. *Curr. Opin. Cell Biol.* **22,** 795–800 (2010).
12. Ginzberg, M. B., Kafri, R. & Kirschner, M. On being the right (cell) size. *Science (80-.).* **348,** 1245075–1245075 (2015).
13. Wang, P. *et al.* Robust growth of escherichia coli. *Curr. Biol.* **20,** 1099–1103 (2010).
14. Osella, M., Nugent, E. & Cosentino Lagomarsino, M. Concerted control of Escherichia coli cell division. *Proc. Natl. Acad. Sci. U. S. A.* **111,** 3431–5 (2014).
15. Godin, M. *et al.* Using buoyant mass to measure the growth of single cells. *Nat. Methods* **7,** 387–90 (2010).
16. Iyer-Biswas, S. *et al.* Scaling laws governing stochastic growth and division of single bacterial cells. *Proc. Natl. Acad. Sci.* 1403232111- (2014). doi:10.1073/pnas.1403232111
17. Di Talia, S. *et al.* The effects of molecular noise and size control on variability in the budding yeast cell cycle. *Nature* **448,** 947–51 (2007).
18. Soifer, I. *et al.* Single-cell analysis of growth in budding yeast and bacteria reveals a common size regulation strategy. *Curr. Biol.* **26,** 356–361 (2016).
19. Tzur, A., Kafri, R., Lebleu, V. S., Lahav, G. & Kirschner, M. W. Cell growth and size homeostasis in proliferating animal cells. *Science* **325,** 167–71 (2009).
20. Park, K. *et al.* Measurement of adherent cell mass and growth. *Proc. Natl. Acad. Sci. U. S. A.* **107,** 20691–6 (2010).
21. Sung, Y. *et al.* Size homeostasis in adherent cells studied by synthetic phase microscopy. *Proc. Natl. Acad. Sci. U. S. A.* **110,** 16687–92 (2013).
22. Son, S. *et al.* Direct observation of mammalian cell growth and size regulation. *Nat. Methods* **9,** 910–2 (2012).
23. Kafri, R. *et al.* Dynamics extracted from fixed cells reveal feedback linking cell growth to cell cycle. *Nature* **494,** 480–483 (2013).
24. Grilli, J., Osella, M., Kennard, A. S. & Lagomarsino, M. C. Relevant parameters in models of cell division control. *Phys. Rev. E - Stat. Nonlinear, Soft Matter Phys.* **032411,** (2017).
25. Amir, A. Cell Size Regulation in Bacteria. *Phys. Rev. Lett.* **112,** 208102 (2014).
26. Conlon, I. & Raff, M. Differences in the way a mammalian cell and yeast cells coordinate cell growth and cell-cycle progression. *J. Biol.* **2,** 7 (2003).
27. Wallden, M., Fange, D., Gregorsson Lundius, E., Baltekin, Ö. & Elf, J. The synchronization of replication and division cycles in individual E. coli cells (in press). *Cell* **166,** 729–739 (2016).
28. Kennard, A. S. *et al.* Individuality and universality in the growth-division laws of single E. Coli cells. *Phys. Rev. E - Stat. Nonlinear, Soft Matter Phys.* **93,** 1–18 (2016).
29. Taheri-Araghi, S. *et al.* Cell-Size Control and Homeostasis in Bacteria. *Curr. Biol.* **25,** 385–391 (2015).
30. Kiviet, D. J. *et al.* Stochasticity of metabolism and growth at the single-cell level. *Nature* **514,** 376–379 (2014).
31. Nobs, J.-B. & Maerkl, S. J. Long-term single cell analysis of S. pombe on a microfluidic microchemostat array. *PLoS One* **9,** e93466 (2014).
32. Adiciptaningrum, A. *et al.* Stochasticity and homeostasis in the E. coli replication and division cycle. *Sci. Rep.* **5,** 18261 (2015).
