# Supplementary material for: Size control in mammalian cells involves modulation of both growth rate and cell cycle duration
Source: Nat Commun. 2018 Aug 16;9:3275. doi: 10.1038/s41467-018-05393-0 (PMC6095894; doi:10.1038/s41467-018-05393-0)
Supplement: Supplementary file 3 — Description of Additional Supplementary Files [file 41467_2018_5393_MOESM3_ESM.pdf]

Description of Additional Supplementary Files

File Name: Supplementary Movie 1
Description: Long time-lapse acquisition in the FXm device of HT29 expressing hgeminin-mcherry, HeLa expressing hgeminin-GFP and Raji cells. The images show a full field acquired in 10X, dark pillars or area are the pillars that sustain the roof of the chamber at a constant height (see Fig. 1a), the culture medium contains a fluorescent dye (Dextran 10kDa coupled to Alexa488 or Alexa 647). Time is in hrs:min. frame rate is 10min, total acquisition length are 50hrs (HT29-hgem), 19h50 (HeLa-hgem), 39h30 (Raji), scale bar is 100µm.

File Name: Supplementary Movie 2
Description: Long time-lapse acquisition of HeLa expressing MyrPalm-GFP (membrane) and H2B-mcherry (DNA) in microchannels of cross section 102µm2. Time is in hrs:min. frame rate is 5min, total acquisition length is 43h15min, and scale bar is 20µm.

File Name: Supplementary Movie 3
Description: One complete cell cycle of a HT29 cell expressing hgeminin-mcherry in FXM device. Left field shows images obtained with FXm, right field shows hgeminin-mcherry signal. Time is hrs:min, frame rate is 10min, total length of the movie is 18h40, and scale bar is 20µm.

File Name: Supplementary Movie 4
Description: Control (top) and Roscovitine-treated (bottom) HeLa cells expressing hgeminin-GFP in FXm device. For each cell, the movie starts 1 time-point (10min) before cytokinesis of the mother cell and stops 4 time-points (40min) after cytokinesis of the two daughter cells. Time is hrs:min, frame rate is 10min, scale bar is 20µm.
